# Professional Burnout in primary care workforce

**DOI:** 10.1192/j.eurpsy.2025.62

**Published:** 2025-08-26

**Authors:** F. Petrazzuoli

**Affiliations:** WONCA Europe, Ruviano, Italy

## Abstract

**Abstract:**

The so called “silver tsunami” i.e. the unprecedented increase in the number of older patients often affected by multimorbidity is a serious health and socioeconomic concern for modern societies and the interconnected challenges of huge workload, premature burnout, and the shortage of primary care personnel represent a pressing issue for the near future. Addressing the complexity of this issue requires a comprehensive approach due to the scarcity of healthcare professionals in European primary care. The health and well-being of both the primary care workforce and the communities they serve are intrinsically intertwined. Enhancing working conditions is a priority: addressing issues like long hours, heavy workloads, bureaucratic demands, and burnout can enhance the appeal of rural primary care practice, attracting and retaining more professionals. Wonca Europe is actively involved in addressing this issue: in June 2023 at the WONCA Europe 2023 Conference in Brussels with the statement Shortage of European Primary Health Care Workforce and in October 2023 at the 73rd Session of the WHO Regional Committee for Europe, Astana, Kazakhstan. Policymakers, healthcare organizations and professional associations need to collaborate to develop tailored solutions that address the unique challenges faced in each region. By addressing the workload of primary care doctors and other professionals, we can enhance their professional satisfaction, improve patient outcomes, and strengthen primary care as a whole.

**Disclosure of Interest:**

None Declared

